# Effects of ethylenediaminetetraacetic acid, citric acid, and etidronic acid on root dentin mineral content and bond strength of a bioceramic-based sealer: A scanning electron microscopy-energy dispersive spectroscopy study

**DOI:** 10.34172/joddd.40798

**Published:** 2024-03-29

**Authors:** Ahmet Taşan, Esin Özlek

**Affiliations:** Department of Endodontology, Faculty of Dentistry, Van Yuzuncu Yil University, Van, Turkey

**Keywords:** Bioceramic-based sealer, Citric acid, Ethylenediaminetetraacetic acid, Etidronic acid, Root dentin mineral content

## Abstract

**Background.:**

This study assessed the impact of chelating agents, 17% ethylenediaminetetraacetic acid (EDTA), 10% citric acid (CA), and 18% etidronic acid (HEDP), on root dentin mineral content. Scanning electron microscopy-energy dispersive spectroscopy (SEM-EDS) was applied to analyze changes, and the push-out bond strength test was used to measure dentin adhesion of Well-Root ST, a bioceramic root canal sealer.

**Methods.:**

A total of 80 extracted single-rooted lower premolar teeth were included in this study and randomly divided into four groups (n=20): group 1 (17% EDTA), group 2 (10% CA), group 3 (18% HEDP), and group 4 (distilled water, control). After irrigation and drying, SEM-EDS was applied to analyze eight samples from each group at coronal, middle, and apical root regions for mineral content and SEM images. The remaining 12 samples underwent a push-out bond strength test using Well-Root ST sealer and gutta-percha. Kruskal-Wallis and Dunn’s tests were used for statistical analyses.

**Results.:**

Statistically significant differences were found between groups (*P*<0.05). SEM-EDS showed significant differences in C, O, Ca, P, and Ca/P content, with no significant differences in Na and Mg. Push-out bond strength was significantly higher in the 17% EDTA, 10% CA, and 18% HEDP groups compared to the control group, with no significant differences between chelating agents.

**Conclusion.:**

Chelating agents altered root dentin mineral content and improved the adhesive properties of the bioceramic sealer. These findings highlight the importance of considering the selection and use of chelating agents in the clinical practice for root canal treatment.

## Introduction

 An ideal irrigation agent in endodontics should have several desirable properties, including tissue dissolution capability, long-lasting antimicrobial efficacy, non-irritating effects on periapical tissues, effective removal of the smear layer, low surface tension, and compatibility with root canal filling materials without compromising their impermeability.^1^ However, no single irrigation solution currently available fulfills all these requirements. Therefore, it is recommended to use a combination of solutions.^2^ Sodium hypochlorite (NaOCl) is commonly used as an organic solvent, while ethylenediaminetetraacetic acid (EDTA) is used as a chelating agent in endodontics. Nevertheless, when these solutions come into contact with root dentin, they can induce mineral alterations on the dentin surface, leading to unwanted effects such as dentinal tubule erosion.^3^ As a result, research on chelating agents that do not affect the mineral content of root dentin has attracted attention in recent years.^4^

 Citric acid (CA) is a weak organic acid suggested as an alternative to EDTA for smear layer removal during root canal irrigation. CA is available in various concentrations ranging from 1% to 50%, with 10% being the most commonly used concentration.^5^ Etidronic acid (1-hydroxyethylidene-1,1-bisphosphonate; HEDP) is another chelating agent whose use has gradually increased in recent years. HEDP is a chelating agent that can be used in conjunction with NaOCl without compromising its proteolytic or antimicrobial effects. De-Deus et al^6^ compared the efficacy of 9% and 18% concentrations of HEDP for removing the smear layer and reported that the 18% concentration was more effective.

 The calcium silicate-containing materials used in endodontics are biocompatible materials commonly employed in vital pulp treatments, root-end filling, and perforation repairs. These materials are also frequently used as root canal filling sealers. Well-Root ST, a calcium silicate-based sealer, is a bioceramic-based sealer used for root canal filling. It chemically bonds to dentin and promotes the formation of hydroxyapatite crystals on the surface.^7^

 This study evaluated the potential alterations in the mineral content on the root dentin surface caused by different chelating agents (EDTA, CA, and HEDP), as well as the effect of the bioceramic-based Well-Root ST canal sealer on bond strength. The null hypothesis stated that EDTA, CA, and HEDP would not affect the root dentin mineral content or the bond strength of bioceramic-based root canal sealers.

## Methods

 Eighty mandibular premolar teeth with fully formed roots and closed apices were selected for this study. The teeth were examined under a stereomicroscope, and teeth with cracks or fractures were excluded. Teeth without root canal calcification, root resorption, and with a minimum root length of 15 mm were included. Soft and hard tissue deposits on the teeth were removed using a curette, and the teeth were stored in distilled water at room temperature until use. The teeth were decoronated using a low-speed diamond saw under liquid cooling to obtain standardized root lengths of 12 mm. A #15 K-file (Dentsply Maillefer, Ballaigues, Switzerland) was inserted into the root canal, and the working length was determined 1 mm shorter than the apical length. All the samples were shaped using the ProTaper Next rotary file system (Dentsply Maillefer, Ballaigues, Switzerland) up to X4, using the X-Smart Plus endodontic motor (Dentsply Maillefer, Ballaigues, Switzerland). During each instrument change, irrigation was performed using 2 mL of 5.25% NaOCI solution (Imicrly, Konya, Turkey) and a 31G side-vented needle. For the final irrigation, the samples were randomly divided into four groups based on the chelating agent used (n = 20): group 1: 17% EDTA, group 2: 10% CA, group 3: 18% HEDP, and group 4 (control): distilled water. A 10% CA solution was prepared by slowly adding 100 g of CA powder (Koray Chemical, Istanbul, Turkey) and mixing it with a magnetic mixer. An 18% HEDP solution was prepared by adding 700 mL of distilled water to 300 mL of 60% HEDP solution (Koray Chemical, Istanbul, Turkey), bringing the total volume to 1 L and mixing it with a magnetic mixer.^8^

 For the final irrigation, the chelating agents (EDTA, CA, and HEDP) were used in a volume of 5 mL for all samples, and irrigation was performed for 1 minute. Subsequently, all the samples were irrigated with 5 mL of 5.25% NaOCl solution. At the end of the final irrigation, the samples were irrigated with 2 mL of distilled water to minimize any potential long-term effects of the solutions. For SEM-EDS analysis, 32 teeth (8 teeth from each group) that had completed the final irrigation protocol were randomly selected. For the push-out bond strength test, 48 teeth (12 teeth from each group) were randomly selected.

###  SEM-EDS analysis

 Vertical grooves were created on the buccal and lingual surfaces of the root using a thin flame-tipped bur, taking care not to perforate the root canals. The roots were then separated from each other using a chisel and hammer. From each sample, one piece was selected for analysis, while the other piece was not included in the study. The selected samples were left untreated to air dry for 12 hours before SEM-EDS analysis.

 The samples were coated with a thin layer of platinum-palladium using a sputter coater and examined under a scanning electron microscope (SEM) (Carl Zeiss Microscopy GmbH, 07745 Jena, Germany). SEM images were captured at three specific positions: the coronal, middle, and apical regions of the canal walls. These positions were standardized by measuring 3 mm, 6 mm, and 9 mm from the apex of the roots. Reference grooves, 0.5 mm in depth and 0.1 mm in width were created on the outer surface of the root to mark these positions, and corresponding points were marked on the lateral surfaces of the root canals.

###  Push-out bond strength test

 After drying the root canals with paper points (Dentsply), they were filled with bioceramic-based Well Root ST canal sealer (Vericom, Gangwon-Do, Korea) and gutta-percha using the lateral compaction technique. All the samples were stored at 37°C with 100% humidity for one week. Subsequently, three horizontal sections measuring approximately 1 ± 0.2 mm in thickness were obtained from the coronal, middle, and apical regions of each sample using an IsoMet saw (Buehler, Lake Bluff, IL, USA) with water cooling. The thickness of the obtained sections was measured using a digital caliper (Mitutoyo Corp, Kanagawa, Japan).

 The push-out bond strength test was conducted using a universal testing machine (Shimadzu Corporation, Kyoto, Japan). The sections were securely fixed onto an acrylic base with a gap in the middle and connected to the testing machine. A force was applied in the apicocoronal direction using stainless steel tips with diameters of 1.10 mm in the coronal third, 0.8 mm in the middle third, and 0.3 mm in the apical third, corresponding to the diameter of the root canal filling. The maximum failure load was recorded in Newtons and used to calculate the push-out bond strength in megapascals (MPa) using the following formula:

 push-out bond strength (MPa) = N/A

 where N is the maximum load (N), and A is the adhesion area of the root canal filling in mm^2^. The bond surface area of each section was calculated as [π (r_1_ + r_2_)] x [(r_1_ − r_2_)_2_ + h_2_],^1/2^ where π is the constant 3.14, r_1_ and r_2_ are the smaller and larger radii, respectively, and h is the thickness of the section in mm.

###  Statistical analysis

 The data were statistically analyzed using SPSS 23. The analysis results were reported as means ± standard deviations and medians (minimum-maximum) for quantitative data. The normal distribution of the data was assessed using the Shapiro-Wilk test. One-way ANOVA was used to compare the bond strength and mineral content data, which exhibited a normal distribution among the groups. Multiple comparisons were performed using Tukey HSD tests. For the bond strength and mineral content data that did not follow a normal distribution among the groups, the Kruskal-Wallis test was used, and subsequent multiple comparisons were conducted using Dunn’s test. The significance level was set at *P* < 0.05.

## Results

###  SEM-EDS analysis results

 The effects of EDTA, CA, and HEDP on mineral changes in dentin were not significantly different (*P* < 0.001) (Table 1). The control group had the minimum sodium (Na) content (1.146 ± 0.389), and the maximum calcium (Ca) content (39.206 ± 4.62) was found in the control group. Similarly, the CA group had the minimum Na (0.972 ± 0.495) and the maximum Ca (29.022 ± 9.516). In the HEDP group, the minimum Mg (0.653 ± 0.179) and the maximum C (51.392 ± 14.186) were observed.

**Table 1:** Descriptive statistics

	**C**	**O**	**Na**	**Mg**	**Ca**	**P**	**Ca/P**
EDTA	11.369 ± 3.009^a^	35.051 ± 3.816^b^	1.132 ± 0.183	1.073 ± 0.147	37.705 ± 5.165^a^	3.651 ± 1.391^d^	12.791 ± 0.494^ab^
CA	25.818 ± 11.549^b^	32.162 ± 4.519^a^	0.972 ± 0.495	1.407 ± 1.958	29.022 ± 9.516^b^	9.839 ± 3.578^b^	4.547 ± 6.773^b^
HEBP	51.392 ± 14.186^c^	32.86 ± 2.536^ab.^	1.193 ± 0.182	0.653 ± 0.179	8.544 ± 10.412^c^	4.449 ± 3.973^c^	2.791 ± 0.494^ab^
Control	9.339 ± 2.281^a^	32.317 ± 4.251^a^	1.146 ± 0.389	1.257 ± 0.28	9.206 ± 4.62^a.^	16.361 ± 1.717^a.^	2.399 ± 0.161^a^

a-d: There is no difference between methods with the same letter.

**Figure 1:**
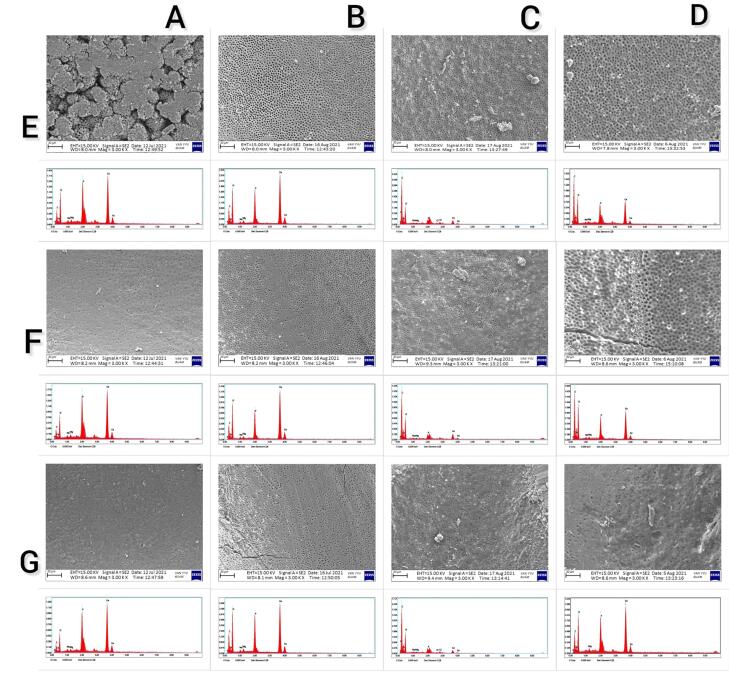
Representative scanning electron microscopic images and energy dispersive spectroscopy of each group ( A. Control, B. EDTA, C. HEBP, and D. CA group; E. Coronal, F. Middle, G. Apical section)

 The effects of EDTA, CA, and HEDP on the changes in the levels of carbon (C), oxygen (O), Ca, phosphorus (P), and Ca/P ratio on the root dentin surface showed statistically significant differences (*P* < 0.001); however, effects on the levels of Na and Mg were not statistically significant (*P* = 0.312 and *P* = 0.058, respectively). The HEDP group exhibited a significantly higher amount of C compared to the CA, EDTA, and control groups. Additionally, there was a statistically significant difference in the amount of C between the CA and control groups (*P* < 0.001); however, no significant difference was found between the EDTA and control groups (*P* > 0.05). The EDTA group showed a significantly higher amount of O compared to the CA and control groups. No statistically significant differences were observed in the amount of O between the CA, HEDP, and control groups and between HEDP and EDTA groups (*P* > 0.05). The HEDP group exhibited a significantly lower amount of Ca compared to the CA and EDTA groups, and the CA group had a significantly lower amount of Ca compared to the control group (*P* < 0.001). However, there were no significant differences between the EDTA and control groups. The P content was significantly lower in the CA, HEDP, and EDTA groups compared to the control group (*P* < 0.001). The Ca/P ratio was significantly higher in the CA group compared to the control group. No significant difference was found between the HEDP and EDTA groups and the control group (*P* > 0.05). Figure 1 shows the SEM images and EDS analysis displaying the findings obtained from a randomly selected sample from the coronal, middle, and apical regions of each group.

###  Bond strength

 Statistically significant differences were observed between the groups (EDTA, CA, HEDP, and control) in terms of bond strength, irrespective of the regions (control, middle, and apical) of the collected data (*P* < 0.001, Table 2). The median bond strength values were 2.04 in the EDTA group, 1.39 in the CA group, 1.54 in the HEDP group, and 1.10 in the control group.

**Table 2:** Bond strength in MPa for each group

		**Mean±SD**	**M (minumum-maximum)**	**Statistical test**	***P***
Coronal	EDTA	1.39 ± 0.51^b^	1.51 (0.58-2.11)	F = 2.852	0.048
	CA	1.12 ± 0.54^ab^	1.18 (0.23-2.11)		
	HEBP	1.26 ± 0.58^ab^	1.28 (0.32-2.10)		
	Control	0.81 ± 0.40^a^	0.71 (0.29-1.66)		
Middle	EDTA	2,34 ± 0.92	2.42 (0.93-3.78)^b^	χ^2^ = 13.622	0.003
	CA	1.77 ± 0.71	1.39 (1.17-3.25)^ab^		
	HEBP	1.70 ± 0.56	1.43 (1.30-3.01)^ab^		
	Control	1.16 ± 0.50	1.03 (0.57-2.34)^a^		
Apical	EDTA	2.73 ± 0.81^b^	2.96 (1.26-3.89)	F = 5.932	0.002
	CA	2.00 ± 0.75^ab^	2.10 (0.59- 2.97)		
	HEBP	1.86 ± 0.77^b^	1.78 (0.66-3.31)		
	Control	1.50 ± 0.57^a^	1.51 (0.69-2.58)		
Total	EDTA	2.15 ± 0.94	2.04 (0.58-3.89)^b^	χ^2^ = 25.479	< 0.001
	CA	1.63 ± 0.76	1.39 (0.23-3.25)^b^		
	HEBP	1.61 ± 0.68	1.54 (0.32-3.31)^b^		
	Control	1.16 ± 0.56	1.10 (0.29-2.58)^a^		

χ^2^: Kruskal Wallis test statistic, F: One-way analysis of variance test statistics, a-b: There is no difference between methods with the same letter SD: Standart deviation.

 Regarding intra-group comparisons, the median bond strength values obtained from the coronal region were 1.39, 1.12, 1.26, and 0.81 in the EDTA, CA, HEDP, and control groups, respectively (*P* = 0.048). In the middle region, the median bond strength values were 2.42 in the EDTA group, 1.39 in the CA group, 1.43 in the HEDP group, and 1.03 in the control group (*P* = 0.003). The median bond strength values obtained from the apical region were 2.73 in the EDTA group, 2.00 in the CA group, 1.86 in the HEDP group, and 1.50 in the control group, with a statistically significant difference (*P* = 0.002).

## Discussion

 The present study investigated the effects of different chelating agents (17% EDTA, 10% CA, and 18% HEDP) on the mineral content of root dentin surfaces. Additionally, the study examined the impact of a bioceramic-based root canal sealer (Well-Root ST) on dentin bond strength. The study’s findings indicated that chelating agents did cause alterations in the mineral content of root dentin, and the canal sealer increased the bond strength. Consequently, the null hypothesis was rejected.

 SEM-EDS analysis was used in this study to assess the effect of chelating agents on the mineral content of root dentin. SEM-EDS analysis is an effective method for identifying and examining surface morphology. This method allows for elemental mapping in specific areas and depths of the tissue. It enables capturing images and determining the mineral content in the selected region simultaneously.^9^ Surface analysis of a specific area was necessary in this study to evaluate the potential relationship between the bond strength and root dentin mineral content. The SEM-EDS method was employed to calculate mean values, determine mineral distribution, and obtain reliable results.

 The results of this study coincide with the findings of Zehnder et al,^10^ who demonstrated that 10% CA solution significantly reduced the amount of Ca⁺⁺ compared to 17% EDTA solution. Similarly, Hennequin and Douillard^11^ reported that CA administration decreased Ca and P content, did not affect Mg content, and increased the Ca/P ratio. Çobankara et al^12^ also reported a statistically significant reduction in Ca⁺⁺ content with both EDTA and CA solutions. Although the 17% EDTA solution in this study decreased the amount of Ca⁺⁺, the difference was not statistically significant. This discrepancy might be attributed to the larger sample size used in the study.

 Akman et al^13^ conducted an SEM-EDS study, reporting that 17% EDTA solution significantly increased the O, Na, and C contents and Ca/P ratio while significantly decreasing the Ca and P contents. On the other hand, they found that 10% CA administration increased the C and Na contents and Ca/P ratio while decreasing the Ca and P ratio. Similarly, 10% CA solutions increased the C content and Ca/P ratio without altering the O and Na contents while decreasing the Ca and P contents. Although some results are consistent between the two studies, there are also differences. These differences might be attributed to the small sample size used in the study by Akman et a.^13^ (18 teeth) and the fact that SEM-EDS analysis was performed only in the coronal triple region of the teeth. In the study by Olcay^14^ on changes in the mineral content of teeth undergoing root canal treatment using SEM-EDS, it was found that the C content increased, the Ca and P content decreased, and the O, Mg, Na, and Ca/P contents did not change significantly in the group that underwent root canal treatment compared to the control group. In the current study, an increase in C content was observed in CA and HEDP groups, no significant difference was found in Na and Mg contents, a decrease in Ca content was observed in the CA and HEDP groups, and a statistically significant decrease in P content was observed in the EDTA, HEDP, and CA groups. The Ca/P ratio did not change significantly in the HEDP and EDTA groups, which is consistent with the findings of the current study. Nogo-Živanović et al^4^ conducted an SEM-EDS study and reported that 17% EDTA solution induced changes in the mineral content compared to the control group, with a statistically significant reduction in P content, which is in line with the results of the present study. Although a decrease in Ca content was observed, it was not statistically significant.

 In contrast to the findings of the current study, Barcellos et al^15^ reported in their SEM-EDS study that 17% EDTA solution did not cause any changes in the mineral content of root dentin. We suggest that this difference in the results could be attributed to the different methodologies of the study. Specifically, Barcellos et al^15^ used a solution volume of 2 mL, while the present study used a volume of 5 mL. It is proposed that using a larger volume of the solution might have led to mineral loss in the dentin.Similarly, using the SEM-EDS method, Lima Nogueira et al^16^ investigated the effect of different irrigation protocols on root dentin. They reported that irrigation protocols containing 17% EDTA resulted in significant mineral loss in terms of Ca and P compared to the control group. Furthermore, protocols containing 9% and 18% HEDP led to a greater loss of Ca and P compared to the 17% EDTA group. These findings partially differ from the results of the current study. We suggest that the differences might stem from variations in the concentrations of the solutions used. Lima Nogueira et al^16^ used 2.5% NaOCl and 9% HEDP solutions, whereas 5.25% NaOCl and 18% HEDP were used in the present study.

 Similar to the present study, Tuncel et al^17^ reported that chelating agents such as 17% EDTA and 9% HEDP increased the bond strength of iRoot SP and AH Plus sealers; however, the difference was not statistically significant. Carvalho et al^18^ found that different chelating agents increased the bond strength of calcium silicate-based sealers, with no significant differences between the chelating agents. Buldur et al^19^ also reported increased bond strength of calcium silicate-based root sealers using chelating agents. In contrast to the findings of the current study, Ballal et al^20^ investigated the bond strength of calcium silicate-based MTA and Biodentine materials with chelating agents in simulated root tip cavities. They reported a significantly lower bond strength in the 17% EDTA group compared to the control group. We believe that this difference might be attributed to the failure of the 17% EDTA solution to effectively remove the smear layer in the apical third of the root canals.^21,22^ Similarly, El-Ma’aita et al^23^ found that 17% EDTA solution significantly reduced the bond strength of calcium silicate-based materials to dentin. This difference can be explained by the larger particle size of calcium silicate-based cements, which might hinder their penetration into dentinal tubules.^24,25^ Donnermeyer et al^26^ reported that a 17% EDTA solution significantly reduced the bond strength of the calcium silicate-based BioRoot RCS sealer. The authors proposed that the decreased calcium content at the interface of the canal patent or deterioration of the calcium silicate fraction in the sealer might prevent the formation of the “mineral infiltration zone” suggested by Atmeh et al.^27^ It is suggested that these factors may negatively affect the bond between the canal sealer and dentin.

 The findings reported by Moon et al^28^ support the idea that removing the smear layer enhances the penetration of root canal filling sealers into dentinal tubules, leading to increased retention of the root canal filling. Similarly, Tuncel et al^17^ reported that the smear layer hinders the bonding of calcium silicate-based root canal sealers to dentin. Calcium silicate-based sealers have been found to form a unique interfacial layer known as the “mineral infiltration zone” within the root dentin wall. This chemical interaction at the dentin interface, combined with micromechanical interactions through tag-like structures, contributes to the adhesion between the sealers and dentin.^27,29^ In the present study, it was observed that the bond strength of the bioceramic-based root canal sealer to dentin was high after removing the smear layer with chelating agents. This finding might be attributed to the improved penetration of the root canal sealer into the exposed dentinal tubules and the subsequent increase in adhesion. Overall, these findings support the importance of smear layer removal in facilitating the bonding and adhesion of root canal sealers to dentin, particularly in the case of calcium silicate-based sealers.

## Conclusion

 In conclusion, this study demonstrated that chelating agents significantly impact the mineral content of root dentin. The analysis revealed an increase in certain minerals and a decrease in others following treatment with chelating agents. All the chelating agents tested, including 18% HEDP, 10% CA, and 17% EDTA, resulted in the dissolution of Ca and P from the root dentin. Additionally, all chelating agents increased carbon (C) content, with the highest increase observed with 18% HEDP, followed by 10% CA and 17% EDTA. Furthermore, when comparing the effect of EDTA, CA, and HEDP solutions to the control group, all chelating agents increased the bond strength of the bioceramic-based Well-Root ST root canal sealer. Based on these findings, HEDP and CA solutions might be considered alternative irrigation agents to EDTA. However, it is important to note that further research is needed to explore the efficacy and safety of these alternative solutions in clinical practice.

 Overall, this study sheds light on the mineral content alternations caused by chelating agents in root dentin and highlights the potential of HEDP and CA solutions in improving the bond strength of bioceramic-based root canal sealers. Continued investigation in this area will contribute to a better understanding of irrigation techniques and aid in the development of effective strategies for root canal treatment.

## Competing Interests

 All authors declare that they have no conflicts of interest.

## Ethical Approval

 All procedures performed in studies involving human participants were in accordance with the ethical standards of the institutional and/or national research committee and with the 1964 Helsinki Declaration and its later amendments or comparable ethical standards. The study was approved by the Institutional Review Board and the Ethics Committee of the University (2021/06-06).

## Funding

 No funding was obtained for this study.

## Informed Consent

 All patients signed an informed consent form after being informed about the study’s objectives, procedures, benefits, and potential risks.
